# Ca^2+^ signals mediated by bradykinin type 2 receptors in normal pancreatic stellate cells can be inhibited by specific Ca^2+^ channel blockade

**DOI:** 10.1113/JP271468

**Published:** 2015-11-08

**Authors:** Oleksiy Gryshchenko, Julia V. Gerasimenko, Oleg V. Gerasimenko, Ole H. Petersen

**Affiliations:** ^1^ Medical Research Council Group, Cardiff School of BiosciencesCardiff UniversityCardiffCF10 3AXWalesUK; ^2^ Bogomoletz Institute of PhysiologyKiev01024Ukraine

## Abstract

**Key points:**

- Bradykinin may play a role in the autodigestive disease acute pancreatitis, but little is known about its pancreatic actions.
- In this study, we have investigated bradykinin‐elicited Ca^2+^ signal generation in normal mouse pancreatic lobules.
- We found complete separation of Ca^2+^ signalling between pancreatic acinar (PACs) and stellate cells (PSCs). Pathophysiologically relevant bradykinin concentrations consistently evoked Ca^2+^ signals, via B2 receptors, in PSCs but never in neighbouring PACs, whereas cholecystokinin, consistently evoking Ca^2+^ signals in PACs, never elicited Ca^2+^ signals in PSCs.
- The bradykinin‐elicited Ca^2+^ signals were due to initial Ca^2+^ release from inositol trisphosphate‐sensitive stores followed by Ca^2+^ entry through Ca^2+^ release‐activated channels (CRACs). The Ca^2+^ entry phase was effectively inhibited by a CRAC blocker.
- B2 receptor blockade reduced the extent of PAC necrosis evoked by pancreatitis‐promoting agents and we therefore conclude that bradykinin plays a role in acute pancreatitis via specific actions on PSCs.

**Abstract:**

Normal pancreatic stellate cells (PSCs) are regarded as quiescent, only to become activated in chronic pancreatitis and pancreatic cancer. However, we now report that these cells in their normal microenvironment are far from quiescent, but are capable of generating substantial Ca^2+^ signals. We have compared Ca^2+^ signalling in PSCs and their better studied neighbouring acinar cells (PACs) and found complete separation of Ca^2+^ signalling in even closely neighbouring PACs and PSCs. Bradykinin (BK), at concentrations corresponding to the slightly elevated plasma BK levels that have been shown to occur in the auto‐digestive disease acute pancreatitis *in vivo*, consistently elicited substantial Ca^2+^ signals in PSCs, but never in neighbouring PACs, whereas the physiological PAC stimulant cholecystokinin failed to evoke Ca^2+^ signals in PSCs. The BK‐induced Ca^2+^ signals were mediated by B2 receptors and B2 receptor blockade protected against PAC necrosis evoked by agents causing acute pancreatitis. The initial Ca^2+^ rise in PSCs was due to inositol trisphosphate receptor‐mediated release from internal stores, whereas the sustained phase depended on external Ca^2+^ entry through Ca^2+^ release‐activated Ca^2+^ (CRAC) channels. CRAC channel inhibitors, which have been shown to protect PACs against damage caused by agents inducing pancreatitis, therefore also inhibit Ca^2+^ signal generation in PSCs and this may be helpful in treating acute pancreatitis.

## Key points

- Bradykinin may play a role in the autodigestive disease acute pancreatitis, but little is known about its pancreatic actions.
- In this study, we have investigated bradykinin‐elicited Ca^2+^ signal generation in normal mouse pancreatic lobules.
- We found complete separation of Ca^2+^ signalling between pancreatic acinar (PACs) and stellate cells (PSCs). Pathophysiologically relevant bradykinin concentrations consistently evoked Ca^2+^ signals, via B2 receptors, in PSCs but never in neighbouring PACs, whereas cholecystokinin, consistently evoking Ca^2+^ signals in PACs, never elicited Ca^2+^ signals in PSCs.
- The bradykinin‐elicited Ca^2+^ signals were due to initial Ca^2+^ release from inositol trisphosphate‐sensitive stores followed by Ca^2+^ entry through Ca^2+^ release‐activated channels (CRACs). The Ca^2+^ entry phase was effectively inhibited by a CRAC blocker.
- B2 receptor blockade reduced the extent of PAC necrosis evoked by pancreatitis‐promoting agents and we therefore conclude that bradykinin plays a role in acute pancreatitis via specific actions on PSCs.

**Abbreviations**
2‐APB: 2‐aminoethoxydiphenylborate
ACE: angiotensin‐converting enzyme
AM: acetoxymethyl
BK: bradykinin
B2: bradykinin type 2
CCK: cholecystokinin
CCKR: cholecystokinin receptor
CFTR: cystic fibrosis transmembrane conductance regulator
CRAC: Ca^2+^ release‐activated Ca^2+^ channel
DAPI: 4′,6‐diamidino‐2 phenylindole
ER: endoplasmic reticulum
IP_3_: inositol trisphosphate
IP_3_R: inositol trisphosphate receptor
PAC: pancreatic acinar cell
PI: propidium iodide
PSC: pancreatic stellate cell

## Introduction

It has been known for more than 40 years that pancreatic enzyme secretion is regulated by Ca^2+^ signals in the pancreatic acinar cells (PACs) and that the primary event is acetylcholine‐ (ACh) or cholecystokinin‐ (CCK) elicited intracellular Ca^2+^ release followed by Ca^2+^ entry (Case & Clausen, 1973; Matthews *et al*. 1973; Kondo & Schulz, 1976; Petersen & Ueda, 1976). The ACh‐evoked intracellular Ca^2+^ release is mediated by inositol trisphosphate (IP_3_) (Streb *et al*. 1983; Wakui *et al*. 1990), whereas the CCK‐elicited Ca^2+^ release is mediated by nicotinic acid adenine dinucleotide phosphate (Cancela *et al*. 2000; Gerasimenko *et al*. 2015). As high K^+^ depolarization of the PAC membrane does not evoke enzyme secretion or Ca^2+^ movement (Argent *et al*. 1971; Matthews *et al*. 1973), the Ca^2+^ entry does not occur through voltage‐gated channels (Petersen, 1992), but is due to opening of Ca^2+^ release‐activated Ca^2+^ (CRAC) channels (Parekh & Putney, 2005; Gerasimenko *et al*. 2013). The acinar Ca^2+^ signals also regulate acinar fluid secretion via Ca^2+^‐activated Cl^−^ and K^+^ channels (Petersen, 1992; Park *et al*. 2001). However, the major component of pancreatic fluid secretion is contributed by the ducts and this is regulated by secretin‐elicited intra‐ductal cyclic AMP formation controlling cystic fibrosis transmembrane conductance regulator (CFTR) channels (Argent, 2006), whose openings are also regulated by the Cl^−^ concentration in the luminal fluid (Broadbent *et al*. 2015).

The often fatal human disease acute pancreatitis, in which the pancreas digests itself and its surroundings, is initiated by excessive intracellular Ca^2+^ release in the PACs followed by excessive Ca^2+^ entry, mostly elicited by combinations of alcohol and fatty acids or by bile acids (Petersen & Sutton, 2006; Gerasimenko *et al*. 2014). Pancreatitis‐inducing agents also inhibit ductal fluid and bicarbonate secretion, exacerbating the disease (Pallagi *et al*. 2011; Maleth *et al*. 2015).

We have extensive knowledge of the mechanisms generating Ca^2+^ signals in the PACs, which has been built up over many decades (Petersen & Tepikin, 2008; Gerasimenko *et al*. 2014). In contrast, the more recently discovered pancreatic stellate cells (PSCs) (Watari *et al*. 1982), located in the peri‐acinar space – with elongated processes around the base of the acinus – have received much less attention and it has essentially been the functional properties of cultured cells that have been investigated (Apte *et al*. 1998, 2012; Bachem *et al*. 1998; Wells & Crawford, 1998). The prevailing, and so far unchallenged, view has been that normal PSCs are quiescent and the focus in all published studies has been on the activation of PSCs and their role – in the activated state – in chronic pancreatitis and pancreatic cancer (Bachem *et al*. 1998; Wells & Crawford, 1998; Sherman *et al*. 2014). Activation of PSCs – during pancreatic injury or culturing of quiescent PSCs – induces proliferation as well as secretion of extracellular matrix components, thereby playing an important role in the fibrosis that occurs in chronic pancreatitis and pancreatic cancer (Sherman *et al*. 2014). Work on the so‐called quiescent PSCs in culture has shown that they can generate substantial cytosolic Ca^2+^ signals in response to stimulation with high concentrations of the blood pressure‐lowering nona‐peptide bradykinin (BK) and some other substances (Won *et al*. 2011).

Because BK has long been known as an important player in inflammatory disease, including acute pancreatitis (Ryan *et al*. 1964; Hirata *et al*. 2002), we have now investigated BK‐induced Ca^2+^ signal generation, and its underlying mechanism, in normal PSCs in their normal microenvironment. We find that normal PSCs are much more sensitive to BK than PSCs in culture, generating substantial Ca^2+^ signals in response to a BK concentration as low as 100 pm, with maximal effect at 1 nm, orders of magnitude lower than what has been observed in cultured cells (Won *et al*. 2011). This has important implications, as the threshold for activating normal PSCs (100 pm) is close to the normal plasma level of BK (40–70 pm) (Blais *et al*. 1999; Hirata *et al*. 2002) and any increase in the plasma or tissue BK levels, which occurs under several conditions – including acute pancreatitis and use of angiotensin‐converting‐enzyme (ACE) inhibitors (Liu *et al*. 1997; Blais *et al*. 1999; Tsutsumi *et al*. 1999; Hirata *et al*. 2002; Su, 2014) – would therefore elicit Ca^2+^ signals in PSCs. We have explored the Ca^2+^ signalling events and their underlying mechanisms in normal PSCs and show that BK activates bradykinin type 2 (B2) receptors, which causes primary Ca^2+^ release from internal stores. This effect can be abolished by phospholipase C inhibition or blockade of IP_3_ receptors (IP_3_Rs). Following the initial intracellular Ca^2+^ release, there is opening of conventional CRAC channels (Parekh & Putney, 2005; Parekh, 2010) and we show that a recently employed CRAC channel inhibitor, which was protective against the destruction of PACs evoked by agents inducing pancreatitis (Gerasimenko *et al*. 2013, 2014), also reduces BK‐induced Ca^2+^ signal generation in PSCs. We show that B2 receptor blockade protects against the necrosis evoked by pancreatitis‐inducing agents and suggest that the protective effect of CRAC channel blockade against pancreatitis may in part be due to inhibition of Ca^2+^ signal generation in PSCs. Overall, our new data indicate that the so far generally accepted notion of normal PSCs being quiescent is potentially misleading as they are in fact exquisitely sensitive to relatively small changes in BK concentrations found *in vivo*.

## Methods

### Ethical approval

All procedures were carried out in accordance with UK Home Office regulations.

### Preparation of pancreatic lobules

**Figure 1:**
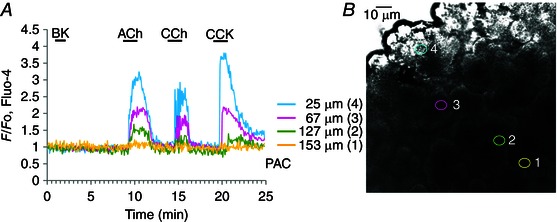
**Pancreatic lobule preparation: penetration of applied substances into deep layers** Experiment illustrating the difficulties of attaining the full concentration level deep inside the pancreatic tissue of substances applied to the surface of a lobule. *A*, [Ca^2+^]_i_ traces obtained from PACs at different depths from the surface of the lobule (signposted by the colour coding shown). Responses to ACh (10 μm), carbachol (CCh) (10 μm) and CCK (10 nm) are shown. *B*, transmitted light image of the lobule from which the recordings shown in *A* were obtained. The different recording positions are indicated using the colour coding shown in *A*.

Pancreatic lobules and big clusters were isolated from the pancreas of adult C57Bl/6 male mice. Animals were killed according to UK Schedule 1 regulations. The pancreas was rapidly dissected, transferred to collagenase Na‐Hepes‐based solution (Sigma, Poole, UK) and incubated at 37°C for 5–6 min. After digestion, the tissue was kept in Na‐Hepes‐based extracellular media, containing (in mm): NaCl, 140; KCl, 4.7; Hepes (KOH), 10; MgCl_2_, 1; glucose 10; CaCl_2_ 1; pH 7.2. Pancreatic lobules were then incubated with fluorescent dye following the manufacturer's description. All experiments were carried out with freshly prepared pancreatic lobules, attached to the coverslip of the perfusion chamber at room temperature (23°C). Penetration of various substances deep into the pancreatic lobule was highly dependent on the distance from the surface (Fig. 1 *A*, *B*). It was therefore necessary, in several cases, to use relatively high concentrations (up to 5‐fold higher than would have been necessary in experiments on isolated cells or very small cell clusters) and relatively long pre‐incubation times.

### [Ca^2+^] measurements in intact cells

Intact cells were loaded with 5 μm Fluo‐4 acetoxymethyl ester (AM), for 10 min at room temperature. Cells were transferred to a flow chamber and perfused with the Na‐Hepes‐based extracellular solution as described above. Cells were visualized using a Leica SP5 MPII two‐photon confocal microscope, with a 63× 1.2 NA objective lens. Fluo‐4 was excited with a 488 nm Ar laser, at 1–4% power, and emitted light was collected at 510–580 nm. Generally, a series of images was recorded at 512×512 pixel resolution (at a speed of 0.3 frames s^−1^), and analysed using Leica Confocal Software (Leica, Mannheim, Germany). Fluorescence signals were plotted as *F*/*F*_0_ (*F*_0_ is the initial level of fluorescence). Statistical analysis was performed using ANOVA or Student's *t*‐test.

### Measurements of necrosis level in pancreatic lobules

Pancreatic lobules were exposed to 350 mm ethanol, or 500 μm palmitoleic acid ethyl ester (POAEE) or 0.5% sodium choleate for 2 h in the presence or absence of 0.5 μm or 10 μm WIN 64338. Ethanol‐ or bile‐induced pancreatic necrosis was visualized by staining the lobules with propidium iodide (PI) and compared with the control necrosis level (without any treatment). Simultaneously lobules were stained with the nuclear dye Hoechst 33342 to enable cell counting. Both stainings were performed according to the manufacturer's protocols. Fluorescence of Hoechst 33342 and PI was recorded using confocal microscopy, with a 63× 1.2 NA objective lens (excitations 355 and 543 nm; and emissions 390–480 and 570–650 nm, respectively).

### Immunocytochemistry

Immunocytochemistry in pancreatic lobules and big clusters was performed as described by Lur *et al*. (2009) with some modifications. Following blocking with 1% BSA and 10% goat serum, the isolated pancreatic lobules were incubated for 1 h at room temperature with primary antibody (CCK‐AR) in 5% goat serum in PBS. The pancreatic lobules were subsequently incubated with CruzFluor (CFL) 594‐conjugated secondary antibody for 30 min at room temperature. Cells were attached to the glass coverslips covered with poly‐l‐lysin. For immunochemical staining with desmin antibody, pancreatic lobules were fixed with 4% paraformaldehyde followed by permeabilization with Triton X100, blocking and incubation with primary (desmin) and then with secondary (CFL) antibodies as described above.

### Reagents

Chemicals, unless otherwise indicated, were obtained from Sigma or Calbiochem (Merck, UK). BK, R‐715 and WIN64338 were purchased from Tocris Biosciences (Bristol, UK). Fluo‐4 AM, PI and Hoechst 33342 were purchased from Invitrogen (Life Technologies, Carlsbad, CA, USA). Antibodies against CCK‐A (CCK1) receptor (sc‐16172), desmin antibody (sc‐7559) and donkey anti‐goat IgG‐CFL 594 (sc‐362275) were from Santa Cruz Biotechnology (Santa Cruz, CA, USA).

## Results

### Separate Ca^2+^ signalling events in neighbouring PSCs and PACs

**Figure 2:**
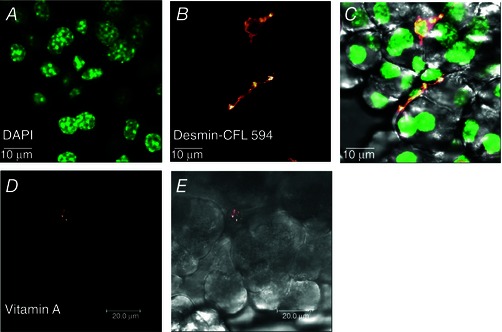
**Identification of PSCs in lobule preparation** *A*, localization of nuclei is shown by staining with DAPI. *B*, staining with desmin antibody, visualized with conjugated secondary antibody IgG‐CruzFluor 594 (CFL 594). *C*, overlay of transmitted light image with DAPI and desmin antibody (*n* = 6). *D*, visualization of lipid droplets containing vitamin A by multiphoton intrinsic fluorescence (*n* = 6). *E*, transmitted light image of the pancreatic lobule shown in *D*.

It has been shown that desmin is a good marker for PSCs (Apte *et al*. 1998) and we found that immunochemical staining with desmin antibody identified small elongated cells situated at the periphery of the dominant acinar cells (Fig. 2 *A–C*). The presence of vitamin A in lipid droplets is another characteristic of PSCs (Bachem *et al*. 1998) and we could visualize this by intrinsic multiphoton fluorescence (Fig. 2 *D*, *E*).

**Figure 3:**
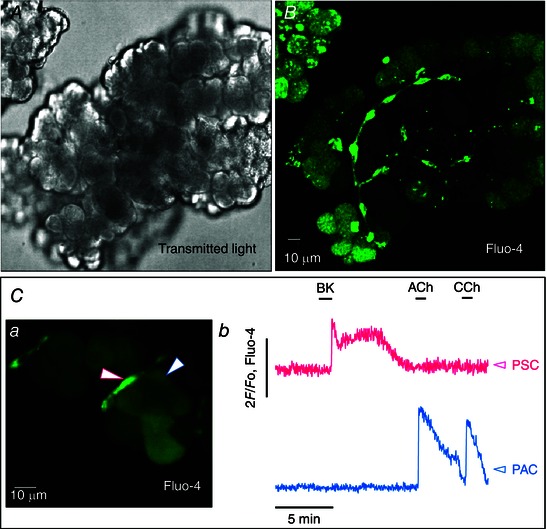
**Complete separation of Ca^2+^ signalling in neighbouring PSCs and PACs** *A*, transmitted light image of live mouse pancreatic lobule. *B*, fluorescence image of the same lobule (Fluo‐4). The small elongated cells with long processes (PSCs) are much better loaded with Fluo‐4 than the larger PACs. *C*, [Ca^2+^]_i_ measurements in neighbouring PSCs and PACs in a small cell cluster. *a*, fluorescence image of the live small cell cluster from which recordings were made. A highly fluorescent PSC is signposted by the white arrow outlined in red, whereas the much less fluorescent neighbouring PAC is signposted by the white arrow with blue outline. *b*, [Ca^2+^]_i_ traces from the two cells signposted in *a*. Red trace from the PSC and blue trace from the PAC. BK (1 nm) evokes a typical biphasic Ca^2+^ signal in the PSC but not in the neighbouring PAC, whereas ACh (10 μm) and CCh (10 μm) evoke Ca^2+^ signals in the PAC but not in the PSC.

Cells situated in the position characteristic of desmin‐containing cells (Fig. 2 *C*) took up the Ca^2+^‐sensitive fluorescent probe Fluo‐4 much more avidly than PACs (Fig. 3 *A–C*). In large lobules stained with Fluo‐4 (Fig. 3 *A*, *B*), it was often possible to observe PSCs as bright ‘strings’, whereas in smaller cell clusters, they typically appeared as individual bright cells at the periphery of acinar units (Fig. 3 *Ca*).

**Figure 4:**
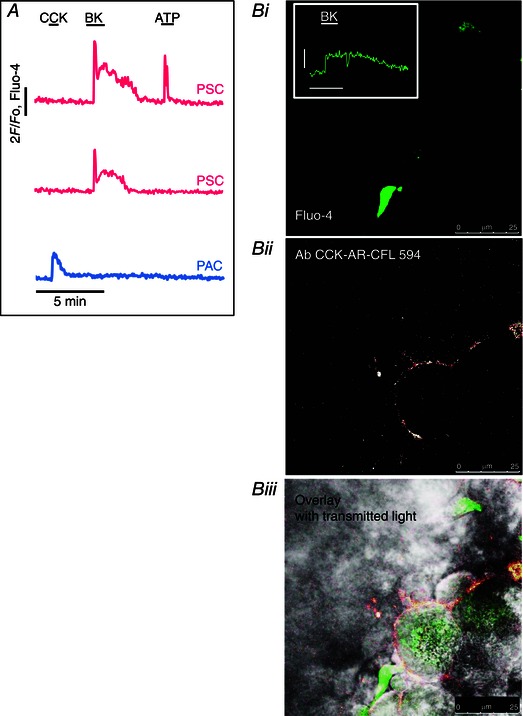
**BK, but not CCK, evokes Ca^2+^ signal in PSCs** *A*, simultaneous [Ca^2+^]_i_ traces from two PSCs and one PAC in the same cell cluster. CCK (10 nm) only evoked a Ca^2+^ signal in the PAC but not in the two PSCs, whereas BK (1 nm) elicited Ca^2+^ signals in both PSCs but not the PAC. ATP (100 μm) evoked a response in one PSC but not in the other and not in the PAC. *B*, immunostaining of CCK receptors in a pancreatic lobule. *i*, a PSC identified by bright staining with Fluo‐4. The response to BK (5 nm) is shown in the inset (amplitude scale bar 1 *F*/*F*_0_, time scale bar 2 min). *ii*, visualization of CCK1 receptors by antibody against the CCK1 receptor with subsequent application of secondary antibody (CFL 594). *iii*, overlay of *i* and *ii* showing the localization of CCK1 receptors on the surface of PACs, but not the PSC.

Cultured PSCs produce cytosolic Ca^2+^ signals in response to a micromolar concentration of BK (Won *et al*. 2011). In our experiments, normal PSCs in their normal micro‐environment typically responded to a short‐lasting exposure to a much lower BK concentration (1 nm) with a sharp transient rise in [Ca^2+^]_i_, quickly followed by a longer lasting (plateau) phase of elevated [Ca^2+^]_i_ (Figs 3 *Cb* and 4 *A*). Neighbouring PACs never displayed any change in [Ca^2+^]_i_ during the BK‐induced PSC Ca^2+^ signals (>100 experiments) (Figs 3 *Cb* and 4 *A*), indicating lack of functional BK receptors on PACs and lack of direct communication between neighbouring PACs and PSCs. The typical, and physiologically important, PAC stimulants ACh and CCK evoked Ca^2+^ signals in PACs (Petersen & Tepikin, 2008), which were not transmitted to the neighbouring PSCs (*n* = 14 and 17, respectively) (Figs 3 *Cb* and 4 *A*). ATP (100 μm) elicited Ca^2+^ signals in a proportion of PSCs (41 of 107 cells), but failed to do so in many others (Fig. 4 *A*). When ATP evoked a [Ca^2+^]_i_ rise in a PSC, it was never transmitted to the neighbouring PAC (Fig. 4 *A*).

Phillips *et al*. (2010) have suggested that CCK‐evoked release of ACh from PSCs could in turn activate PACs and they proposed that this could be the normal mechanism for CCK‐elicited secretion from human PACs. However, we did not find any evidence for this hypothesis as CCK never evoked any Ca^2+^ signals in PSCs in our preparations (*n* = 17; at [CCK] = 10 nm) and Ca^2+^ signals in PSCs consistently failed to be transmitted to neighbouring PACs (Fig. 4 *A*). Furthermore, although a fluorescent antibody to CCK1 receptors clearly identified the presence of CCK1 receptors on PACs, it failed to do so with regard to PSCs (four experiments) (Fig. 4 *B*).

### BK evokes Ca^2+^ signals in PSCs at pico‐ and low nanomolar concentrations

**Figure 5:**
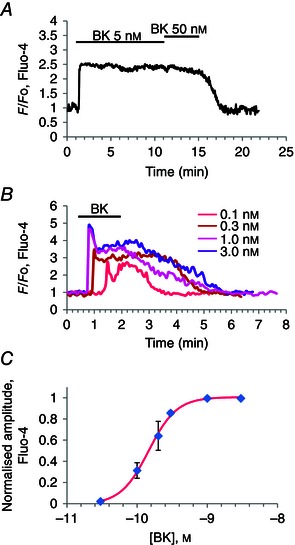
**BK concentration–response relationship** *A*, [Ca^2+^]_i_ trace from PSC showing that 5 nm BK evokes maximal sustained response, which is not enhanced by increasing the BK concentration to 50 nm. *B*, family of [Ca^2+^]_i_ traces all obtained from the same PSC, showing responses to BK concentrations from 0.1 to 3 nm. *C*, BK concentration–response (amplitude of plateau phase) relationship. Half‐maximal response is at a BK concentration of 200 pm. A very similar relationship was obtained for the initial peak response.

It seemed important to explore the levels of BK needed to evoke Ca^2+^ signals (Fig. 5) in relation to what is known about BK concentrations in plasma. From measurements in humans and rats, it is known that normal plasma BK concentrations are in the range 40–70 pm (Blais *et al*. 1999; Hirata *et al*. 2002). However, in a bile duct obstruction model of acute pancreatitis induced in rats, it has been shown that the BK plasma concentration rose to ∼140 pm (Hirata *et al*. 2002). As the BK release in acute pancreatitis primarily comes from the pancreas, the local (intra‐pancreatic) BK levels could be higher (Ryan *et al*. 1964; Schachter, 1969). We found that a BK concentration as low as 100 pm (*n* = 14) could elicit PSC Ca^2+^ signals and that maximal responses were obtained at 1 nm (*n* = 9, in the series of experiments represented by Fig. 5 *C*). Overall, in the course of this investigation, BK responses to 1 nm have been observed in >100 experiments. At the low BK concentrations we used, there was no sign of desensitization (Fig. 5 *A*). Within the time frame of our protocols, the [Ca^2+^]_i_ elevation was maintained as long as the stimulus was maintained (*n* = 19) (Fig. 5 *A*). It would therefore appear that normal PSCs are sufficiently sensitive to BK to be able to sense relatively small increases in the surrounding BK level.

**Figure 6:**
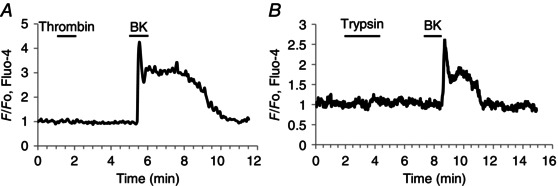
**Thrombin and trypsin do not evoke Ca^2+^ signals in PSCs** *A*, thrombin (10 mU ml^−1^) does not evoke a Ca^2+^ signal in a PSC, but a subsequent BK application (1 nm) evokes the usual response. *B*, trypsin (20 μm) fails to evoke a Ca^2+^ signal in a PSC, but a subsequent BK application (1 nm) does so. The apparently delayed and somewhat smaller response to BK (although still within the normal range) in *B* (as compared to *A*) is probably due to the fact that the cell cluster in this case was relatively large and the PSC deep in the tissue (see Fig. 1).

Although the PSCs in our lobule preparation were much more sensitive to BK than those previously studied in culture (Won *et al*. 2011), they do have some characteristics in common with quiescent PSCs in culture which, unlike activated PSCs, do not respond to trypsin and thrombin. As seen in Fig. 6 there was no response to thrombin (*n* = 6) (Fig. 6 *A*) or trypsin (*n* = 18) (Fig. 6 *B*) in cells that responded to BK with a clear Ca^2+^ signal.

### The BK‐elicited Ca^2+^ signals in PSCs are due to activation of B2 receptors

**Figure 7:**
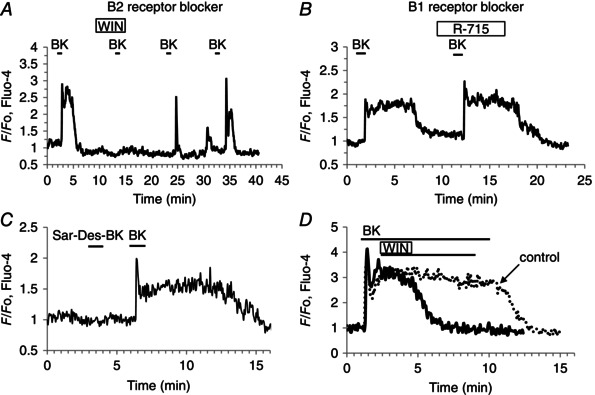
**BK receptor pharmacology** *A*, the B2 receptor antagonist WIN64338 (10 μm) reversibly and completely blocked the BK‐ (1 nm) elicited Ca^2+^ signal, both peak (*P* < 0.000003) and plateau (*P* < 0.00004). *B*, the B1 receptor antagonist R‐715 (10 μm) failed to block the BK‐ (1 nm) elicited Ca^2+^ signal. *C*, the B1 receptor agonist Sar‐des‐Arg‐BK (Sar‐Des‐BK) (1 μm) does not evoke a Ca^2+^ signal in a PSC, whereas a subsequent application of BK (1 nm) elicits such a signal. *D*, the B2 receptor antagonist WIN64338 applied after the initial BK‐evoked Ca^2+^ spike reduces markedly the length of the plateau phase.

The BK receptor sub‐type responsible for generating Ca^2+^ signals in PSCs has not previously been investigated. We employed the B2 receptor blocker WIN64338 (Hu *et al*. 2004) and showed that this agent reversibly blocked BK‐elicited Ca^2+^ signal generation (*n* = 27) (Fig. 7 *A*). In contrast, the B1 receptor blocker R‐715 (Abdouh *et al*. 2008) failed to inhibit BK‐induced Ca^2+^ signalling (*n* = 8) (Fig. 7 *B*). Furthermore, the specific B1 agonist Sar‐des‐Arg‐BK did not evoke any changes in [Ca^2+^]_i_ of PSCs (*n* = 8) (Fig. 7 *C*). It would appear that the plateau phase of the BK‐induced response to some extent depends on continued B2 receptor activation, because application of WIN64338 immediately after the initial BK‐elicited Ca^2+^ spike shortened the duration of the plateau phase (Fig. 7 *D*).

**Figure 8:**
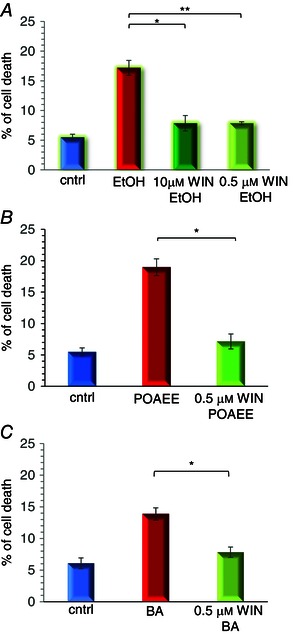
**The extent of PAC death elicited by pancreatitis‐inducing agents is markedly reduced by B2 receptor blockade** *A*, treatment of pancreatic lobules with ethanol (350 mm) for 2 h at room temperature increased significantly the percentage of PAC necrosis from the control level of 5.47 ± 0.54% (*n* = 7) to 17 ± 1% (*n* = 7) and this was significantly reduced to 7.85 ± 1.3% (*n* = 3) by 10 μm WIN64338 and to 7.84 ± 0.25% (*n* = 4) by 0.5 μm WIN64338 (*P = 0.0025 and **P = 0.00043, respectively, >1500 cells in each experimental group). *B*, treatment of pancreatic lobules with POAEE (500 μm) for 2 h at room temperature increased significantly the percentage of PAC necrosis from the control level of 5.46 ± 1.25% (*n* = 3) to 18.96 ± 1.76% (*n* = 3) and this was reduced to 7.15 ± 0.67% (*n* = 3) by 0.5 μm WIN64338 (*P = 0.003, >990 cells in each experimental group). *C*, treatment of pancreatic lobules with 0.5% sodium choleate for 2 h at room temperature also increased significantly the percentage of PAC necrosis from the control level of 6.1 ± 0.85% (*n* = 3) to 14 ± 1% (*n* = 3) and this was significantly reduced to 7.8 ± 0.8% (*n* = 3) by 0.5 μm WIN64338 (*P = 0.0087, >1600 cells in each experimental group). Necrosis was measured by staining of lobules with PI. Cell count was performed using nuclear staining with Hoechst 33342. Bars represent ±SEM.

As a previous study showed that BK levels increase in acute pancreatitis and that blockade of B2 receptors attenuated the cellular changes underlying the development of acute pancreatitis evoked by obstruction of the pancreatico‐biliary duct in rats (Hirata *et al*. 2002), our results raise the possibility that B2 receptor‐mediated Ca^2+^ signals in PSCs contribute to the negative outcome of this disease. We therefore tested whether B2 receptor blockade could protect against cellular changes relevant to the development of acute pancreatitis, which is most frequently related to alcohol abuse or biliary disease (Petersen & Sutton, 2006). In alcohol‐related acute pancreatitis, the pancreas is exposed not only to alcohol, but also to fatty acid ethyl esters (non‐oxidative combinations of alcohol and fatty acids), which have been shown to be particularly effective releasers of intracellular Ca^2+^ (Criddle *et al*. 2006; Gerasimenko *et al*. 2009). In biliary disease, the pancreas will be exposed to high concentrations of bile acids, which have also been shown to be effective liberators of stored Ca^2+^ (Voronina *et al*. 2002; Gerasimenko *et al*. 2006). We therefore tested the effects of B2 receptor blockade on the level of necrosis elicited by alcohol, POAEE and a bile acid mixture. As seen in Fig. 8, B2 receptor blockade markedly reduced the extent of acinar cell necrosis induced by either a high alcohol concentration (350 mm), POAEE (500 μm) or bile acids (0.5% sodium choleate: a crude ox bile extract which contains the sodium salts of taurocholic, glycocholic, deoxycholic and cholic acids).

### BK‐elicited Ca^2+^ signals in PSCs are primarily due to release of Ca^2+^ from internal stores, but is followed by store‐operated Ca^2+^ entry

**Figure 9:**
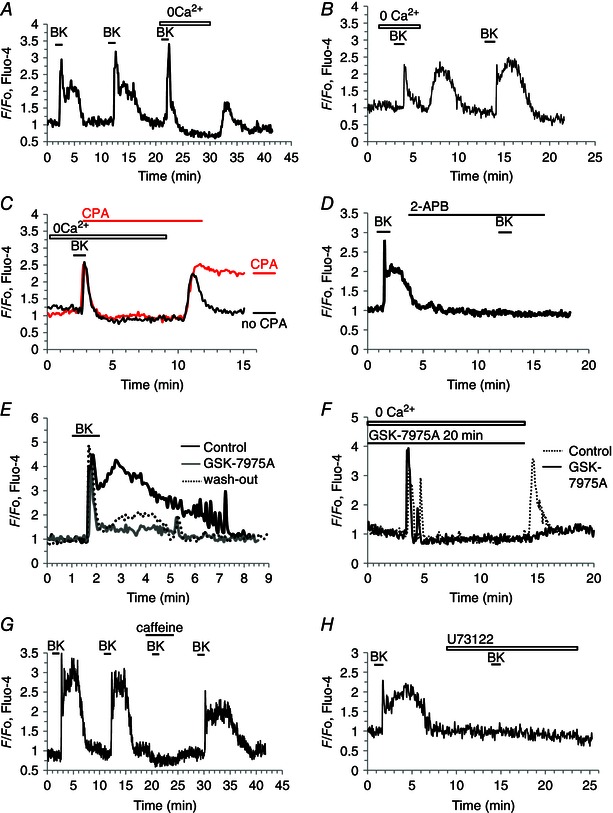
**Mechanisms of BK‐elicited Ca^2+^ signal generation in PSCs** *A* and *B*, removal of external Ca^2+^ has no effect on the initial [Ca^2+^]_i_ rise evoked by BK (1 nm, *P *> 0.7), but abolishes the following plateau phase (*P* < 0.0003). Readmitting external Ca^2+^, in the absence of BK stimulation, causes a transient rise in [Ca^2+^]_i_. *C*, when the SERCA pump inhibitor CPA (20 μm) is present, the [Ca^2+^]_i_ rise upon readmission of external Ca^2+^ is prolonged. *D*, 2‐APB (100 μm) (IP_3_R antagonist and inhibitor of CRAC channels) blocks Ca^2+^ signalling elicited by BK (1 nm). *E*, the CRAC channel blocker GSK‐7975A (10 μm) reduces markedly the plateau phase of the BK‐ (1 nm) elicited response (*P* < 0.0015). Washout of GSK‐7975A partially restored the response (*P* <  0.009). *F*, GSK (10 μm) does not inhibit the initial BK‐elicited Ca^2+^ signal occurring in the absence of external Ca^2+^ but prevents the [Ca^2+^]_i_ rise normally occurring upon external Ca^2+^ readmission. *G*, caffeine (30 mm) reversibly blocks BK‐ (1 nm) elicited Ca^2+^ signal. *H*, the phospholipase C inhibitor U73122 (30 μm) abolished Ca^2+^ signal generation elicited by BK (1 nm), both peak (*P* < 0.0002) and plateau (*P* < 0.00001).

As shown in Figs 3, 4, 5, the BK‐elicited Ca^2+^ signals consist of a brief transient rise in [Ca^2+^]_i_, followed by a prolonged plateau phase of elevated [Ca^2+^]_i_. Acute removal of external Ca^2+^ did not reduce the initial phase of BK‐evoked Ca^2+^ signals, but eliminated the following plateau phase (Fig. 9 *A*, *B*). Re‐admission of Ca^2+^ resulted in a transient increase in [Ca^2+^]_i_ and enabled a subsequent BK application to evoke a normal response (Fig. 9 *A*, *B*). We tested the ability of PSCs to generate store‐operated Ca^2+^ entry, by using the now ‘classical’ protocol for assessing this phenomenon (Fig. 9 *C*), employing cyclopiazonic acid (CPA) to block the Ca^2+^ pumps in the endoplasmic reticulum (ER). As shown in Fig. 9 *B* and *C*, re‐admission of external Ca^2+^ after a period of external Ca^2+^ deprivation, and BK stimulation, resulted in a transient rise in [Ca^2+^]_i,_ but, if the Ca^2+^ re‐admission occurred after and during a period of continued ER Ca^2+^ pump inhibition, the [Ca^2+^]_i_ elevation was sustained (Fig. 9 *C*). In this situation, Ca^2+^ entering store‐operated Ca^2+^ channels cannot be taken up into the ER, but will only slowly be extruded by the plasma membrane Ca^2+^ pumps.

We tried to block the store‐operated Ca^2+^ entry pharmacologically. 2‐APB, a well‐known, but relatively unspecific, blocker of CRAC channels (Parekh & Putney, 2005), abolished BK‐induced Ca^2+^ signal generation (Fig. 9 *D*), which may be due to blockade of IP_3_Rs (Ma *et al*. 2000). We also used a more specific CRAC channel blocker, GSK‐7975A, which has recently been shown to block CRAC channel currents in PACs (Gerasimenko *et al*. 2013). In these experiments, we were able to show that GSK‐7975A reversibly blocked the plateau phase of the BK‐induced [Ca^2+^]_i_ elevation, without affecting the initial spike (*n* = 9)(Fig. 9 *E*). GSK‐7975A also blocked the Ca^2+^ entry normally occurring when external Ca^2+^ was re‐admitted after a period of external Ca^2+^ deprivation (*n* = 14) (Fig. 9 *F*).

Finally, we examined the mechanism underlying the initial Ca^2+^ spike in response to BK stimulation. Having established that it is due to release of Ca^2+^ from internal stores (Fig. 9 *A*), we tested the hypothesis that this Ca^2+^ liberation occurs through IP_3_Rs. It is now well stablished that caffeine inhibits opening of IP_3_Rs (Wakui *et al*. 1990; Ehrlich *et al*. 1994; Foskett *et al*. 2007). As seen in Fig. 9 *G*, caffeine did reversibly block BK‐elicited Ca^2+^ signalling (*n* = 29). BK probably activates phospholipase C, as U73122 (Bleasdale *et al*. 1990; Smith *et al*. 1990) blocked the ability of BK to evoke Ca^2+^ signals (*n* = 14) (Fig. 9 *H*).

## Discussion

Our results demonstrate for the first time that BK, in concentrations close to those measured in normal plasma (Blais *et al*. 1999; Hirata *et al*. 2002), elicits substantial cytosolic Ca^2+^ signals in normal PSCs in their normal micro‐environment and therefore cast doubt on the hitherto prevailing concept of the quiescent PSC (Apte *et al*. 1998, 2012; Sherman *et al*. 2014).

Given that an important role for BK in the development of acute pancreatitis was proposed by Ryan *et al*. (1964), that there is an increase in the plasma level of BK in acute pancreatitis (Hirata *et al*. 2002) and that a number of studies have shown that pharmacological blockade of B2 receptors is helpful in suppressing the cellular changes in several pancreatitis disease models (Griesbacher & Lembeck 1992; Griesbacher *et al*. 1993; Hoffmann *et al*. 1996; Bloechle *et al*. 1998; Hirata *et al*. 2002), it would seem important to identify the target for the action of BK in the pancreas and its mechanism of action.

Won *et al*. (2011) demonstrated that BK – in high (micromolar) concentrations – evoked transient Ca^2+^ signals in quiescent and activated PSCs in culture. We focused our attention on the effect of low quasi‐physiological concentrations of BK on normal PSCs in their normal micro‐environment, the mechanism of action and its consequence. Our results show that whereas BK consistently evokes bi‐phasic Ca^2+^ signals in PSCs, it consistently fails to do so even in closely neighbouring PACs (Figs 3 and 4). We have therefore now identified a specific cellular site for the action of BK, which may partly or fully explain its importance in pancreatitis. Furthermore, we have established that the BK‐elicited Ca^2+^ signals in PSCs are due to activation of B2 receptors, providing a plausible explanation for the suppressive effect of B2 blockade on the development of acute pancreatitis (Griesbacher & Lembeck 1992; Griesbacher *et al*. 1993; Hoffmann *et al*. 1996; Bloechle *et al*. 1998; Hirata *et al*. 2002). We have also shown that PAC necrosis elicited by alcohol, POAEE or bile can be significantly reduced by B2 receptor blockade (Fig. 8).

We failed to observe any effects of CCK on PSCs, although this hormone evoked its usual effect (Petersen & Tepikin, 2008) on neighbouring PACs (Fig. 4 *A*). Therefore, our results do not provide support for the hypothesis that CCK acting on PSCs will increase ACh secretion from these cells, which in turn would activate PACs (Phillips *et al*. 2010). We worked on normal mouse pancreatic tissue, whereas Phillips *et al*. (2010) studied ACh secretion from cultured human PSCs. Cultured PSCs clearly have different properties from normal PSCs, because in the normal mouse pancreas we have observed Ca^2+^ signal generation in PSCs in response to 0.1 nm BK (Fig. 5), whereas micromolar BK concentrations were required to obtain such responses in cultured mouse PSCs (Won *et al*. 2011). With regard to the mechanism of CCK action in the human pancreas, the simplest hypothesis remains direct action on PACs, as shown in isolated acinar cell clusters from human pancreas (Murphy *et al*. 2008).

Although the initiating event elicited by pancreatitis‐inducing agents in PACs is Ca^2+^ release from intracellular stores (Gerasimenko *et al*. 2014), we have previously shown that the cellular damage only happens if there is secondary Ca^2+^ entry from the extracellular fluid (Raraty *et al*. 2000). We have recently shown that the CRAC channel blocker GSK‐7975A markedly inhibits the store‐operated Ca^2+^ entry that sustains the [Ca^2+^]_i_ elevation in PACs evoked by a pancreatitis‐inducing agent (Gerasimenko *et al*. 2013, 2014), a finding recently confirmed by Voronina *et al*. (2015). We have previously shown that the CRAC channel blockade by GSK‐7975A provides effective protection of the PACs from alcohol‐related intracellular protease activation and necrosis (Gerasimenko *et al*. 2013) and these results have also very recently been confirmed in a study employing three different *in vivo* mouse models of acute pancreatitis (Wen *et al*. 2015). The BK‐elicited Ca^2+^ signal generation in PSCs is due to initial release of Ca^2+^ from internal stores followed by activation of Ca^2+^ entry via store‐operated channels. Our new results show that GSK‐7975A is also effective in reducing the plateau phase of the BK‐elicited [Ca^2+^]_i_ elevation in PSCs (Fig. 9 *E*). Given that B2 receptor blockade protects against pancreatitis‐like cellular changes (Fig. 8), the inhibitory effect of GSK‐7975A on BK responses in PSCs may represent an additional potential benefit of treating cases of severe acute pancreatitis with a CRAC channel blocker. The inhibitory action of GSK‐7975A on the BK‐induced plateau elevation of [Ca^2+^]_i_ in PSCs may well have contributed to the impressive protective effects of this agent against acute pancreatitis *in vivo* (Wen *et al*. 2015).

Our new data are also relevant when considering the now widespread use of ACE inhibitors in the treatment of hypertension because ACE inhibitors inhibit the breakdown of BK, causing an increase in the tissue and plasma levels of BK (Israili & Hall, 1992; Liu *et al*. 1997; Tsutsumi *et al*. 1999; Su, 2014). Previous studies have shown that use of ACE inhibitors is associated with a significantly increased risk of developing acute pancreatitis (Tilkemeier & Thompson, 1988; Eland *et al*. 2006), but the mechanism was unknown. As we have now shown that any increase in the BK level will elicit Ca^2+^ signals in the PSCs via B2 receptors and that blockade of these receptors protects against acute pancreatitis, it is likely that Ca^2+^ signal generation in PSCs mediated by BK is at least in part responsible for the increased risk of developing acute pancreatitis during ACE inhibitor treatment.

## Additional information

### Competing interests

None declared.

### Author contributions

Conception and design of the experiments: O.H.P., O.V.G., O.G. and J.V.G. Collection, analysis and interpretation of data: O.G., J.V.G. and O.V.G. Drafting the article or revising it critically for important intellectual content: O.H.P., J.V.G., O.V.G. and O.G.

### Funding

This work was supported by Medical Research Council Programme Grant MR/J002771/1. O.H.P. is a Medical Research Council Professor (G19/22/2).

**Translational Perspective**

Our work indicates that bradykinin‐elicited Ca^2+^ signals in pancreatic stellate cells may influence negatively the outcome of acute pancreatitis. We tested the hypothesis that blockage of bradykinin type 2 (B2) receptors would offer protection against the pancreatic acinar cell necrosis caused by pancreatitis‐inducing agents, such as alcohol, fatty acid ethyl esters or bile acids. The results showed that specific B2 receptor blockade markedly reduced the extent of necrosis observed after treatment with ethanol, POAEE or a mixture of bile acids. This suggests that B2 receptor blockade in the early stage of acute pancreatitis may be helpful in reducing the severity of the disease. We also show that the bradykinin‐elicited sustained elevation of the cytosolic Ca^2+^ concentration in pancreatic stellate cells can be inhibited by a specific inhibitor of Ca^2+^ release – activated Ca^2+^ (CRAC) channels. We have recently shown that CRAC inhibition in PACs offers remarkable protection against necrosis induced by fatty acid ethyl esters. In the intact pancreas, CRAC inhibition would reduce excessive Ca^2+^ signal generation both in the acinar cells (brought about by, for example, fatty acid ethyl esters) and in the stellate cells (brought about by bradykinin). Both effects would be beneficial. Our work therefore indicates that combined treatment with a CRAC inhibitor and a B2 receptor – blocking agent should be tested *in vivo* as a potentially useful therapy against acute pancreatitis.
